# Functional Studies of the Yeast Med5, Med15 and Med16 Mediator Tail Subunits

**DOI:** 10.1371/journal.pone.0073137

**Published:** 2013-08-22

**Authors:** Miriam Larsson, Hanna Uvell, Jenny Sandström, Patrik Rydén, Luke A. Selth, Stefan Björklund

**Affiliations:** 1 Department of Medical Biochemistry and Biophysics, Umeå University, Umeå, Sweden; 2 Department of Statistics, Umeå University, Umeå, Sweden; 3 Mechanisms of Transcription Laboratory, Clare Hall Laboratories, Cancer Research UK London Research Institute, South Mimms, United Kingdom; Università degli Studi di Milano, Italy

## Abstract

The yeast Mediator complex can be divided into three modules, designated Head, Middle and Tail. Tail comprises the Med2, Med3, Med5, Med15 and Med16 protein subunits, which are all encoded by genes that are individually non-essential for viability. In cells lacking Med16, Tail is displaced from Head and Middle. However, inactivation of *MED5*/*MED15* and *MED15*/*MED16* are synthetically lethal, indicating that Tail performs essential functions as a separate complex even when it is not bound to Middle and Head. We have used the N-Degron method to create temperature-sensitive (ts) mutants in the Mediator tail subunits Med5, Med15 and Med16 to study the immediate effects on global gene expression when each subunit is individually inactivated, and when *Med5/15* or *Med15/16* are inactivated together. We identify 25 genes in each double mutant that show a significant change in expression when compared to the corresponding single mutants and to the wild type strain. Importantly, 13 of the 25 identified genes are common for both double mutants. We also find that all strains in which *MED15* is inactivated show down-regulation of genes that have been identified as targets for the Ace2 transcriptional activator protein, which is important for progression through the G1 phase of the cell cycle. Supporting this observation, we demonstrate that loss of Med15 leads to a G1 arrest phenotype. Collectively, these findings provide insight into the function of the Mediator Tail module.

## Introduction

Regulation of transcription in eukaryotes by RNA polymerase II (Pol II) is controlled by factors that affect chromatin packing, chromatin structure and by regulatory proteins (activators and repressors) that bind to specific DNA sequences in promoters. In the latter process, the Mediator co-activator complex is required to convey signals from the promoter-bound regulatory proteins to the Pol II transcription machinery [1], [2]. Mediator is an essential and evolutionarily conserved protein complex that is required for transcription of almost all protein encoding genes in eukaryotic cells. It was first identified in budding yeast, and it has since been identified in higher eukaryotes such as *S. pombe*, *C. elegans*, human and *A. thaliana* [3]–[6]. These studies showed that the function and overall structure of Mediator is evolutionarily conserved. Mediator in *S.cerevisiae* comprises 25 different subunits and a combination of genetics, biochemistry and structure biology has revealed that it is composed of three modules; Head, Middle, and Tail [7]–[11]. In addition, Mediator can also associate with a separate kinase module comprising Cdk8, cyclin C, Med12 and Med13. The head module interacts directly with Pol II and stimulates basal, unregulated transcription [1], [2], [12]–[14]. Tail is suggested to make direct interactions with promoter-bound transcriptional regulators and is located most distal from Pol II, where it is bridged to the Middle module via the Med14 subunit [7], [15]. The Cdk8 module is more loosely associated and reversibly associates with Mediator to regulate the Mediator–Pol II interaction [7]–[11], [16] to control transcription initiation and reinitiation.

Several lines of evidence indicate that Tail is important for activation of transcription and numerous reports have identified tail subunits as targets for different transcriptional regulatory proteins (see ref [17] for a review). However, there are also reports on direct interactions between transcriptional regulators and subunits of the Head and Middle modules [18]–[20]. Three-dimensional reconstruction from electron micrographs of single Mediator particles isolated from *med16* (*sin4*) mutants shows that the entire tail domain is absent from the purified complex [21]. Since *med16* cells are viable, it was initially difficult to understand how Tail can play such a fundamental role in the transcription activation process given that all tail module subunits are encoded by non-essential genes. Interestingly, it appears that Tail does not have to be physically connected to Head and Middle in order to function properly. In fact, in the *med16* mutant, Tail subunits are present in a distinct protein complex that is separate from Head and Middle [22]. The free tail complex in *med16* comprises Med2, Med3 (Hrs1), and Med15 (Gal11) and can be functionally recruited to the *ARG1* promoter by interaction with the transcriptional activator protein Gcn4. Opposite to the situation in wild type cells, the Head and Middle modules were not recruited to the *ARG1* promoter in *med16* cells upon activation. Recruitment of Tail alone was sufficient to recruit TBP and Pol II to the *ARG1* promoter and resulted in induction of *ARG1* mRNA expression to the same levels as in wild type cells. In contrast, the *ARG1* mRNA levels were markedly de-repressed under non-inducing conditions in the *med16* strain compared to wild type cells. Similar results were obtained in another study describing the isolation of a “core Mediator”-fraction that contained most of the head and middle module subunits, but lacked Tail [23]. This core Mediator complex could activate transcription *in vitro*, but not to the same extent as complete Mediator. In addition, core Mediator combined with complete Mediator showed a lower degree of activation than the complete Mediator alone. Both of these studies suggest that Tail by itself is mainly involved in transcriptional activation, while Middle and Head together predominantly function in transcriptional repression. Further supporting this concept, we have previously reported that the Med2 subunit of the Tail complex is phosphorylated by the Cdk8 kinase on serine 208 and that a mutation of serine 208 in Med2 to an alanine resulted in a dramatic down regulation of expression of genes encoded on the 2 µM plasmid and an upregulation of a limited set of genes [24]. In line with these results, epistasis and expression profile analysis show that *MED2* and also *MED18* act downstream of *CDK8* [25].

In support of an essential role for the Tail complex, it has been shown that double deletions of either *MED5* and *MED15* or *MED15* and *MED16* are synthetically lethal [26], [27]. However, the cause of the synthetic lethality has not been studied in detail. Here we have used the N-Degron method to create temperature-sensitive (ts) mutants in the Mediator tail subunits Med5, Med15 and Med16. We show that conditional and concomitant inactivation of *MED5* and *MED15* or *MED15* and *MED16* results in altered expression of a limited set of genes that partly overlap. Interestingly, several of these genes are involved in meiosis and sporulation. In support of this observation, all strains where *MED15* was inactivated showed cell cycle defects and repression of target genes for the Ace2 activator protein.

## Materials and Methods

### Yeast strains

YKL200 (obtained from Euroscarf) was used as wild type control for the Degron strains. Single-Degron strains (*med5*, *med15* and *med16*) and double-Degron strains (*med5/med15* and *med15/med16*) were created.

### The Degron system

The degron system was used to deplete a specific protein from *S. Cerevisiae* cells and to examine the immediate effects of the depletion. In short we inserted a degron cassette in the chromosomal locus of the *MED5*, *MED15* and *MED16* genes as described below. The native promoters were replaced by the CUP1 promoter, and a so-called “heat-inducible degron” was added to the N-terminus of each protein. The CUP1 promoter was activated at the permissive growth conditions (YPD(/R)Cu-medium, containing CuSO_4_ and glucose (or raffinose), growing at 24°C) due to the presence of copper in the medium and the degron-tag is inactivated due to the low temperature. At the restrictive growth conditions (YPG-medium, lacking CuSO_4_ and glucose (or raffinose), containing galactose, growing at 37°C) expression of each protein was repressed due to lack of copper and degraded by activation of the degron-tag due to the increased temperature. For more details on the degron system see [28].

### Change of selectable marker in pKL187 (Degron strains)

The *KanMX* gene was replaced with the *NatMX* (nourseothricin resistance) from the pAG25 plasmid [29]. pAG25 and pKL187 were digested with NotI (Fermentas) for 12 hours at 37° C over night, and the resulting linearized vector pKLK187 and the natMX fragment was purified (QIAquick Gel Extraction Kit, Qiagen). pKL187 was treated with Shrimp alkaline phosphatase and natMX was ligated (T4 DNA ligase, Invitrogen) into pKL187.

### Creation of Degron strains

Amplification of the Degron cassette and transformation was performed as described in [28]. When clones were selected with nourseothricin 100 µg/ml clonNAT (Hans-Knöll Institute für Naturstoff-Forshung, Jena, Germany) was used. The following primers were used for amplification of the Degron cassette:

Med5 5′:

GGCAGCTAATTGAAACAGTAATCTACAAATATGAGTAAGCTAAACCACCTCATTAAGGCGCGCCAGATCTG

Med5 3′:

TGCCGCTCAGCACATTTTAGTGCTAAGTTGTATACTGATTCTTTTTCCATGGCACCCGCTCCAGCGCCTG

Med15 5`:

GTTAAACCCCATTTTTATAAGCGTATCGTTTCGTATAGTGCCGATTAAGGCGCGCCAGATCTG

Med15 3′:

CGGCATTGGACAGAGTGTCTTTGTCTTGGACAGGAGCAGCAGACATGGCACCCGCTCCAGCGCCTG

Med16 5′:

CCTTCGTTAGTCTCCTTTGTATATTGCCGTTTGCAGGCTCATGATGATATTAAGGCGCGCCAGATCTG

Med16 3′:

GCAATAATACCAGTCTTAGACCAGCTCATTAAATGCTCTCCAAGCATCATGGCACCCGCTCCAGCGCCTG

Handling of the Degron strains and verification of integration was carried out as described [28]. Sequences for primers for verification are available upon request. All PCR-reactions were carried out using Platinum® Pfx DNA polymerase (Invitrogen). Single-Degron strains were created by selection for clonNAT for *med5* and G418 for *med15* and *med16*. Double-Degron strains were obtained with a second round of transformation with an amplified Degron cassette with the other selectable marker and selection for both clonNAT and G418.

### Plate serial dilution of Degron strains

All growth conditions are outlined in [28]. Briefly, colonies of each strain were dissolved in PBS and diluted to approximately 3.3×10^6^ cells/ml. From this dilution three more 10-fold dilutions were made for each strain. 15 µl of each dilution was dropped on to YPDCu-agar (2% peptone, 1% Yeast extract, 2% glucose, 0.1 M CuSO_4_, 2% agar) plates and YPG-agar (2% peptone, 1% Yeast extract, 2% galactose, 2% agar) plates. The plates where incubated for 2 days at 24°C (YPDCu) and 37°C (YPG).

### Growth curves of Degron strains

All growth conditions are outlined in [28]. Briefly, colonies from each strain were dissolved in 100 ml of YPRCu medium (2% peptone, 1% Yeast extract, 2% raffinose, 0.1 M CuSO_4_) and grown over night on a shaker at 24°C. The cell density was measured and the cultures where diluted with fresh YPRCu medium to 5×10^6^ cells/ml. Growth was continued until the cell density reached 10^7^ cells/ml. The cells where then pelleted and dissolved in YPG medium (2% peptone, 1% Yeast extract, 2% galactose). The cells where incubated at 24°C for 35 minutes to induce the expression of the Ubr1 protein, and then pelleted and dissolved in pre-warmed 37°C YPG medium. The cells where then incubated at 37°C on a shaker and harvested at the indicated time points to examine the growth effects of depleting the proteins.

### Western blotting

Proteins were extracted as described in [30] and run on NuPAGE 3–8% Tris-Acetate gels (Invitrogen). The gels were blotted to Hybond™-P membranes (Amersham), blocked in 2% milk in TBST and subjected to primary antibody. Horseradish peroxidase linked secondary antibody was added for detection of primary antibody. The α-Med5, α-Med15 and α-Med16 antibodies are polyclonal antibodies produced in rabbit using synthesized peptides. α-Med16 has been affinity purified against its peptide. The α-cmyc antibody used is mouse monoclonal antibody (clone 9E10).

### Microarray analysis of Degron Strains

All strains were grown in YPRCu at 24°C to approximately 1×10^7^ cells/ml. The media was switched to YPGalactose and growth was continued at 24°C for 35 minutes. The cells were transferred to pre-warmed YPGalactose (37°C) and growth was continued at 37°C for 45 minutes before harvesting. 2 ml of the cultures was used for RNA extraction (RNeasy Mini, Qiagen). The cells were lysed in RLT buffer by bead beating (7×30 seconds, with chilling on ice for one minute before each round of bead beating). On-column DNase treatment was performed according to the manufacturer. RNA was quality controlled, reverse transcribed and labeled before hybridized to Affymetrixs® Yeast Genome 2.0 Arrays. All strains were grown independently twice and two independent data sets were obtained for each strain. The resultant CEL files were normalized using robust multi-array average (RMA) background correction. Genes with an average fold-change > 1.5 were considered differentially expressed. The microarray dataset has been deposited in Gene Expression Omnibus (GEO) under accession number GSE47712.

### RT-PCR

RNA was extracted as described for microarray analysis. RNA concentrations were measured using a NanoDrop, ND-1000 spectrophotometer. 1 µg of RNA was reverse transcribed using an iScript cDNA Synthesis Kit (BioRad). qPCR was carried out using IQ™ Supermix (BioRad). Equal amounts of cDNA and 500 nM primers were used in each reaction in a total volume of 25 µl. PCR reactions and detection was carried out on iCycler IQ PCR thermal cycler (BioRad). All samples were analyzed in triplicates, and the measured concentration of RNA was normalized to the levels of ACT1 mRNA. Sequences for primers are available upon request.

### Flow cytometry

Determination of DNA content in the different yeast mutants at different time points after switching from permissive to restrictive conditions was performed exactly as described [31].

## Results

### Construction of Heat-inducible N-Degron Mutants for med5, med15 and med16

**Figure 1:**
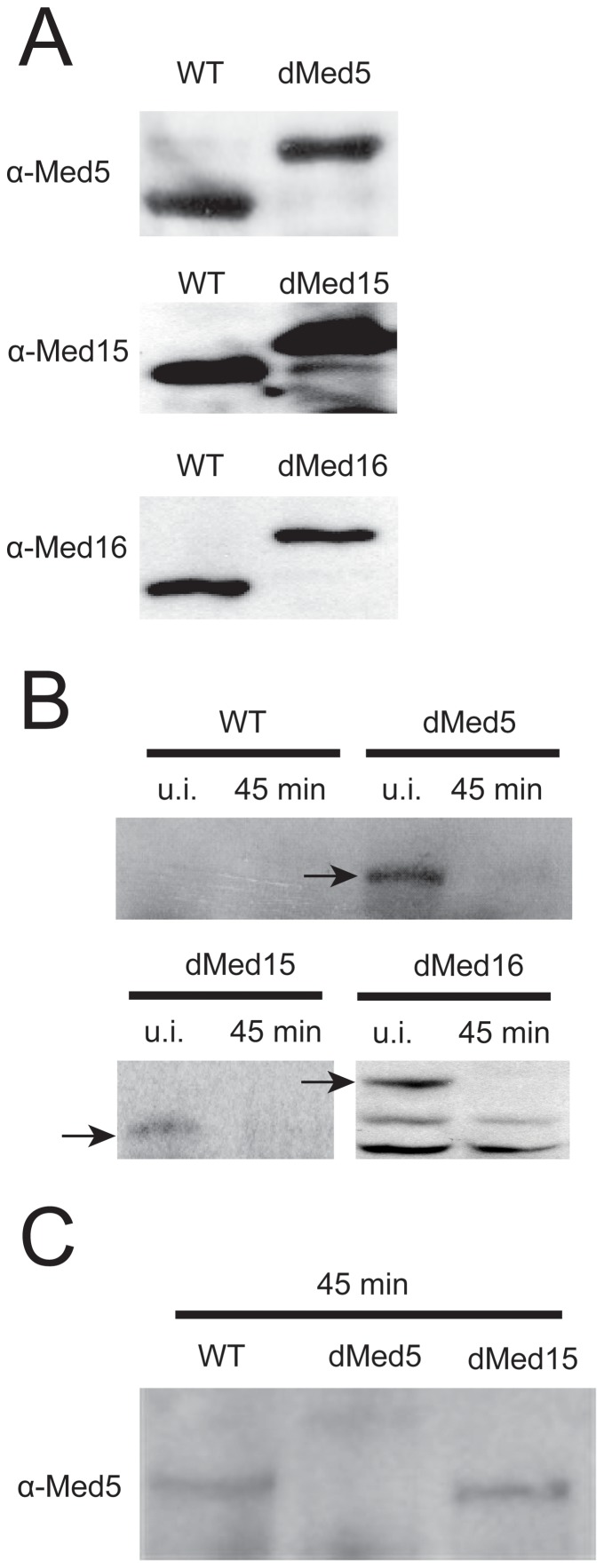
Confirmation of expression and specific, induced degradation of the *MED5*, *MED15* and *MED16* Degron constructs. (A) Crude protein extracts isolated from the wild type strain and each of the *med5*, *med15*, and *med16* Degron strains, grown at the permissive conditions (24°C/YPD/0.1 mM CuSO_4),_ were separated on 10% SDS-PAGE, transferred to PVDF membranes and blotted with α-Med5, α-Med15 and α-Med16 antibodies, respectively. (B) Crude protein extracts were isolated from the wild type strain and each of the *med5*, *med15* and *med16* Degron strains at the permissive conditions (uninduced, u.i.) and 45 minutes after switching to the non-permissive growth conditions. The extracts were separated on 10% SDS-PAGE, transferred to PVDF membranes and blotted with anti c-myc antibodies (specific for the Degron-tag). (C) Specific degradation of Degron-tagged *MED5*. Crude protein extracts were isolated from the wild type strain, the *med5* Degron strain, and the *med15* Degron strain 45 minutes after switching to the restrictive growth conditions. Proteins were separated on 10% SDS-PAGE, transferred to PVDF-membranes and blotted with α-Med5 antibodies.

We adopted the N-Degron system to obtain copper-dependent expression as well as galactose- and temperature-dependent degradation of the Med5, Med15 and Med16 Mediator subunits, either individually or pairwise [28]. All Degron constructs were expressed from their normal chromosomal location under the control of their respective endogenous promoters. In order to study the expression of the Degron-tagged proteins, we prepared whole-cell protein extracts from wild type cells and from each single Degron strain. The extracts were analyzed by Western blotting using antibodies specific for each protein (**Figure 1A**). We found that the Degron-tagged Med5, Med15 and Med16 were all expressed at levels similar to the levels of each protein in the parental wild type strain. When comparing the levels of each Degron-tagged protein, before and after heat-induction, we found that each Degron-tagged protein was efficiently depleted from the extracts already at 45 minutes after heat-induction **(** **Figure 1B** **)**. Finally, we also tested that the heat-degradation of Med5 was specific to the strain expressing the Degron-tagged version. We found that untagged Med5 is stable in both the wild type strain and in the strain that expresses a Degron-tagged version of Med15, but degraded in the strain that expresses the Degron-tagged Med5 (**Figure 1C**). Thus, we conclude that the mutant strains encoding Degron-tagged versions of Med5, Med15 and Med16 function properly.

### Synthetic lethality in strains lacking both Med5 and Med15 or both Med15 and Med16

**Figure 2:**
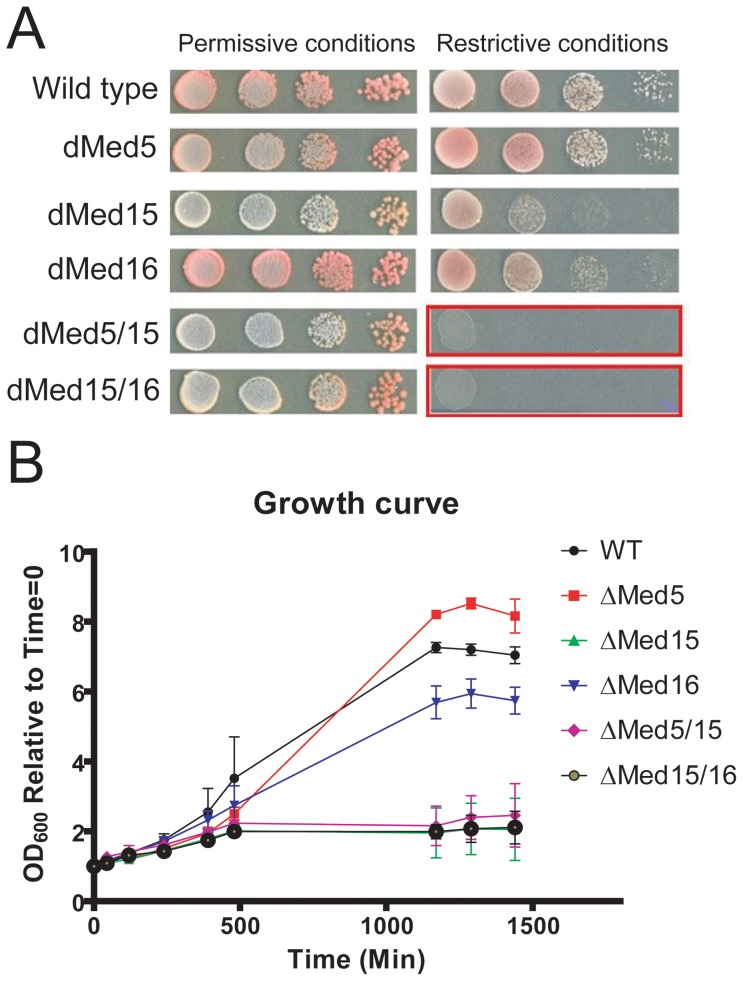
Double deletions of yeast *MED5/MED15* and *MED15/MED16* are synthetically lethal. (A) The strains indicated to the left were plated as 10-fold serial dilutions, 5 µl spots at permissive conditions (24°C, Cu^++^, glucose) and restrictive conditions (37°C, no Cu^++^, galactose) respectively. (B) The strains indicated to the right were grown in liquid media, at restrictive conditions, for 24 hours. OD_600_ was measured at the indicated time points. OD_600_ at 0 hours was set to 1 and the other values where normalized against time = 0 min. The graphs represent the mean of two separate experiments. The graphs represent the mean of two separate experiments, and the error bars represent the standard deviation.

We and others have previously reported that single disruptions of *MED5, MED15* or *MED16* are viable, in contrast to double deletions of *MED5* and *MED15* or *MED15* and *MED16,* which all cause synthetic lethality [26], [27]. However, because the cells carrying the double deletions do not grow it has not previously been possible to study the cause of lethality. In order to test if we could reproduce the synthetic lethality in a controlled, heat-inducible way using the N-Degron system, we grew wild type cells, strains expressing individual Med5, Med15 or Med16 N-Degron constructs and strains that express N-Degron-tagged versions of both Med5/Med15 and Med15/Med16, respectively. The cells were initially grown in YPD at 24°C (permissive conditions), then transferred either to YPG-plates (**Figure 2A**) or liquid YPG-media (**Figure 2B**) and moved to 37°C (restrictive conditions). When the cells were grown on plates, degradation of Med5 alone had little or no effect on growth, while single degradation of Med16 showed a mild growth defect, and single degradation of Med15 showed a more pronounced effect on growth. This limited growth effect for a *med15* mutant has been reported previously [27]. In contrast, we found that both the *med5/med15* and the *med15/med16* double Degron strains were unable to grow on plates at the restrictive conditions (**Figure 2A** **)**. These results indicate that the N-Degron system can be used to reproduce the effects of ts mutants in the tail module that have been created using conventional methods. Similar results were obtained in liquid media (**Figure 2B**).

### Analyses of global gene expression in the single and double Degron strains at the restrictive conditions

**Figure 3:**
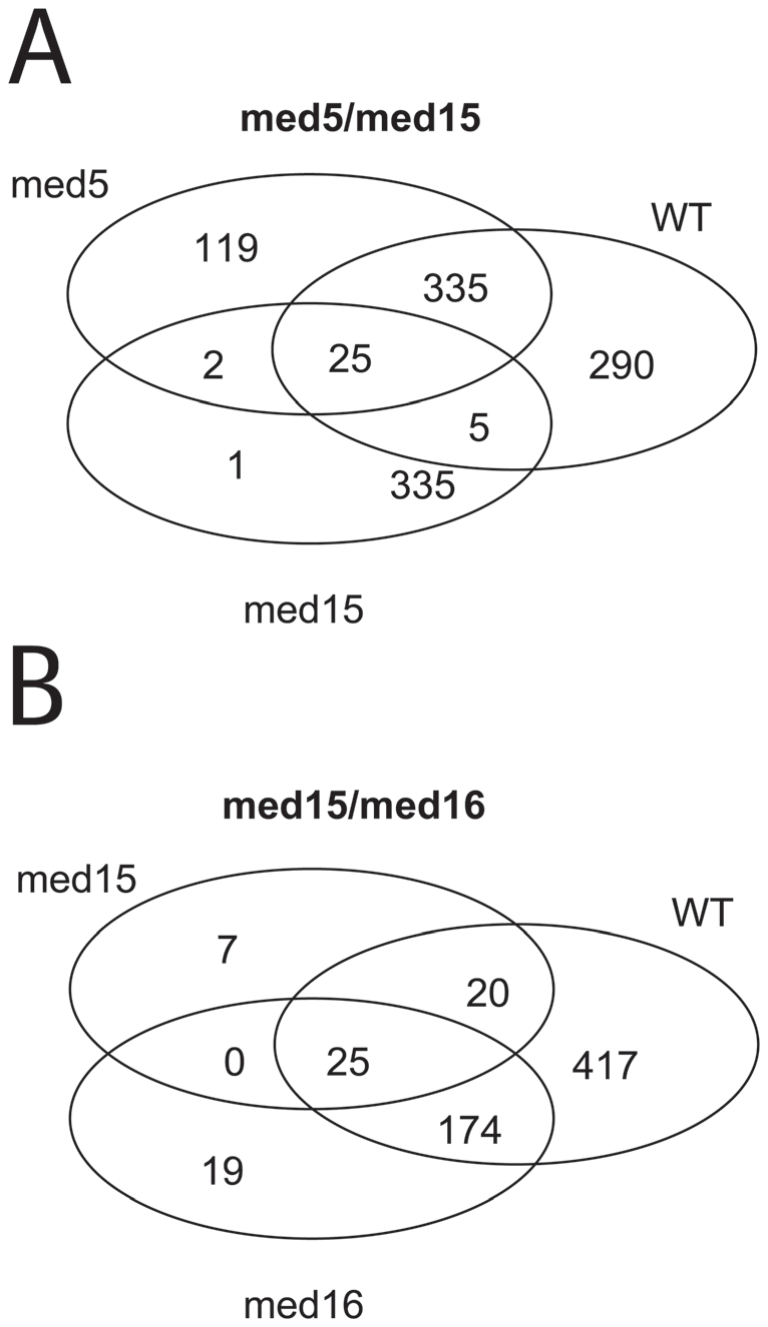
Transcription profile analysis using AffymetrixsYeast Genome 2.0 Array. 25 genes were differently regulated in both of the double-Degron strains (*med5/med15* (A) and *med15/med16* (B)), 45 minutes after induction of degradation, compared to the single Degron (*med5*, *med15* and *med16*) and wild type (Wt) strains, as shown in the Venn diagrams (FDR<0.05 and FC>abs(log2(1.5)).

In order to identify genes that are differentially regulated and therefore could contribute to the synthetic lethality of cells lacking both Med5/Med15 or Med15/Med16, we used Affymetrix microarrays to compare global gene expression in wild type cells and the different Degron strains. We isolated RNA from each strain 45 minutes after changing from the permissive to the restrictive growth conditions in order to minimize possible secondary effects on gene expression that are not directly related to the Degron construct(s). This time point was chosen based on the experiments shown in **Figure 1B**, where the levels of each Degron-tagged protein was undetectable at 45 minutes after switching to the restrictive growth conditions. The results from these experiments including all possible combinations of comparisons of expression between wild type cells, single Degron mutants and double Degron mutants are presented in **Tables 1** **, and S1**. Genes that were differentially expressed in the *med5/med15* strain compared to any individual control strain (WT, *med5*, and *med15* respectively) are depicted as a Venn diagram in **Figure 3A**. Interestingly, the microarray results identified a set of only 25 genes that were uniquely differentially expressed in the *med5/med15* double Degron strain, *i.e.* genes that were differentially expressed when compared to the wild type, single *med5* and single *med15* Degron strains (**Figure 3A** **;** **Table 1**). Considering that the *med15* Degron strain shows the most severe growth defects of all the single Degron mutant strains, it is interesting to note that only 33 genes (25+5+1+2) were uniquely expressed in the *med5/med15* double Degron strain compared to the *med15* single Degron strain. In contrast, 481 genes (25+2+119+335) are differentially expressed when comparing the *med5/med15* double Degron strain with the *med5* single Degron strain and 655 (335+290+25+5) genes are differently expressed when comparing the *med5/med15* double Degron strain with the wild type strain. These results provide an explanation why the single *med15* Degron strain shows a more profound growth defect than the single *med5* strain.

**Table 1:** Genes whose expression is changed 1.5-fold or more in the *med5/med15* double Degron strain compared to either of the wild type, *med5* or *med 15* and in the *med15/med16* double Degron strains compared to either of the wild type, *med15* or *med 16* strain grown at the restrictive conditions.

Gene Name	Gene	Change in Med5/15	Change in Med15/16	Function
*Downregulated*				
YOL155C	HPF1	↓	↓	Haze-protective mannoprotein
YGL158W	RCK1	↓	↓	Protein kinase involved in the response to oxidative stress and in inhibition of meiosis
YHR143W	DSE2	↓	↓	Daughter cell-specific secreted protein with similarity to glucanases degrades cell wall from the daughter side causing daughter to separate from mother
YHR092C	HXT4	↓	↓	High-affinity glucose transporter of the major facilitator superfamily, expression is induced by low levels of glucose and repressed by high levels of glucose
YIL169C	NA	↓	↓	Putative protein of unknown function
YGL028C	SCW11	↓		Cell wall protein with similarity to glucanases; may play a role in conjugation during mating
YER124C	DSE1	↓		Daughter cell-specific protein, may regulate cross-talk between the mating and filamentation pathways
YPL067C	NA	↓		Putative protein of unknown function
YOR178C	GAC1		↓	Regulatory subunit for Glc7p type-1 protein phosphatase (PP1)
YLR377C	FBP1		↓	Fructose-1,6-bisphosphatase, key regulatory enzyme in the gluconeogenesis pathway
YPL036W	PMA2		↓	Plasma membrane H+–ATPase
YAR035W	YAT1		↓	Outer mitochondrial carnitine acetyltransferase
*Upregulated*				
YGL033W	HOP2	↑	↑	Meiosis-specific protein that localizes to chromosomes, preventing synapsis between non-homologous chromosomes and ensuring synapsis between homologs; complexes with Mnd1p to promote homolog pairing and meiotic double-strand break repair
YLR341W	SPO77	↑	↑	Meiosis-specific protein of unknown function required for spore wall formation during sporulation
YOL132W	GAS4	↑	↑	1,3-beta-glucanosyltransferase, involved in spore wall assembly, localizes to the cell wall
YBR040W	FIG1	↑	↑	Integral membrane protein required for efficient mating
YGR273C	IBI1	↑	↑	Putative protein of unknown function, expression downregulated by treatment with 8-methoxypsoralenplus UVA irradiation
YFL012W	NA	↑	↑	Putative protein of unknown function, transcribed during sporulation
YDR042C	NA	↑	↑	Putative protein of unknown function
YGL138C	NA	↑	↑	Putative protein of unknown function
YOR339C	UBC11	↑		Ubiquitin-conjugating enzyme
YDR446W	ECM11	↑		Protein apparently involved in meiosis, may be involved in maintaining chromatin structure
YPL130W	SPO19	↑		Meiosis-specific prospore protein; required to produce bending force necessary for proper assembly of the prospore membrane during sporulation
YOR237W	HES1	↑		Protein implicated in the regulation of ergosterol biosynthesis
YOR242C	SSP2	↑		Sporulation specific protein that localizes to the spore wall, required for sporulation at a point after meiosis II and during spore wall formation
YDR218C	SPR28	↑		Sporulation-specific homolog of the yeast CDC3/10/11/12 family of bud neck microfilament genes; meiotic septin expressed at high levels during meiotic divisions and ascospore formation
YDL114W	NA	↑		Putative protein of unknown function
YOL014W	NA	↑		Putative protein of unknown function
YPL033C	SRL4	↑		Putative protein of unknown function, involved in regulation of dNTP production
YGR177C	ATF2		↑	Alcohol acetyltransferase
YNL111C	CYB5		↑	Cytochrome b5, involved in the sterol and lipid biosynthesis pathways
YHR185C	PFS1		↑	Sporulation protein required for prospore membrane formation at selected spindle poles
YML047C	PRM6		↑	Pheromone-regulated protein
YJR047C	ANB1		↑	Translation elongation factor eIF-5A, previously thought to function in translation initiation; similar to and functionally redundant with Hyp2p; expressed under anaerobic conditions
YKL165C	MCD4		↑	Protein involved in glycosylphosphatidylinositol (GPI) anchor synthesis
YNL130C-A	DGR1		↑	Putative protein of unknown function; dgr1 null mutant is resistant to 2-deoxy-D-glucose
YOR381W-A	NA		↑	Putative protein of unknown function

Similarly, we found that the *med15/med16* double Degron strain showed differential expression of a limited set of 25 genes at the restrictive conditions compared to the wild type, the single *med15* Degron and the single *med16* Degron strains (**Figure 3B** **;** **Table 1**). Importantly, of the 25 genes that are uniquely expressed in each of the double Degron strains, 13 are common to both (**Table 1**). None of these 13 genes are individually essential for viability, which strongly suggests that two or more of these genes encode proteins that together perform essential functions in cells. We therefore decided to look in more detail at the genes affected in both the double Degron strains.

### The double Degron strains show an abnormal up regulation of meiosis and sporulation specific genes

Eight genes were specifically down regulated in the *med5/med15* double Degron strains compared to the control strains (**Table 1** **).** Similarly, nine genes were specifically down regulated in the *med15/med16* double Degron strains compared to the control strains (**Table 1** **).** Of these, five genes were common for both double deletion strains (**Table 1**). Seventeen and sixteen genes, respectively, were up regulated in the *med5/med15* and *med15/med16* double Degron strain compared to the control strains with 8 in common (**Table 1**). When studying these genes in detail we found that as many as 9 of them have been shown to be meiosis specific or involved in sporulation (**Table S2**). This is interesting since the strains used in this study are haploid, and therefore do not go through either meiosis or sporulation. Moreover, we noted that one of the genes found to be down regulated in both Degron strains, *RCK1* (**Table 1**), encodes a protein that is involved in repression of meiosis. It has been shown that deletion of *RCK1* leads to an increased rate of meiosis and sporulation in diploid cells [32]. Since this gene is downregulated in both the *med5/med15* and the *med15/med16* Degron strains it could provide a common explanation for the up regulation of meiosis and sporulation genes in the double Degron strains.

### Importance of the tail module for transcriptional activation of target genes for the ACE2 transcription factor

**Figure 4:**
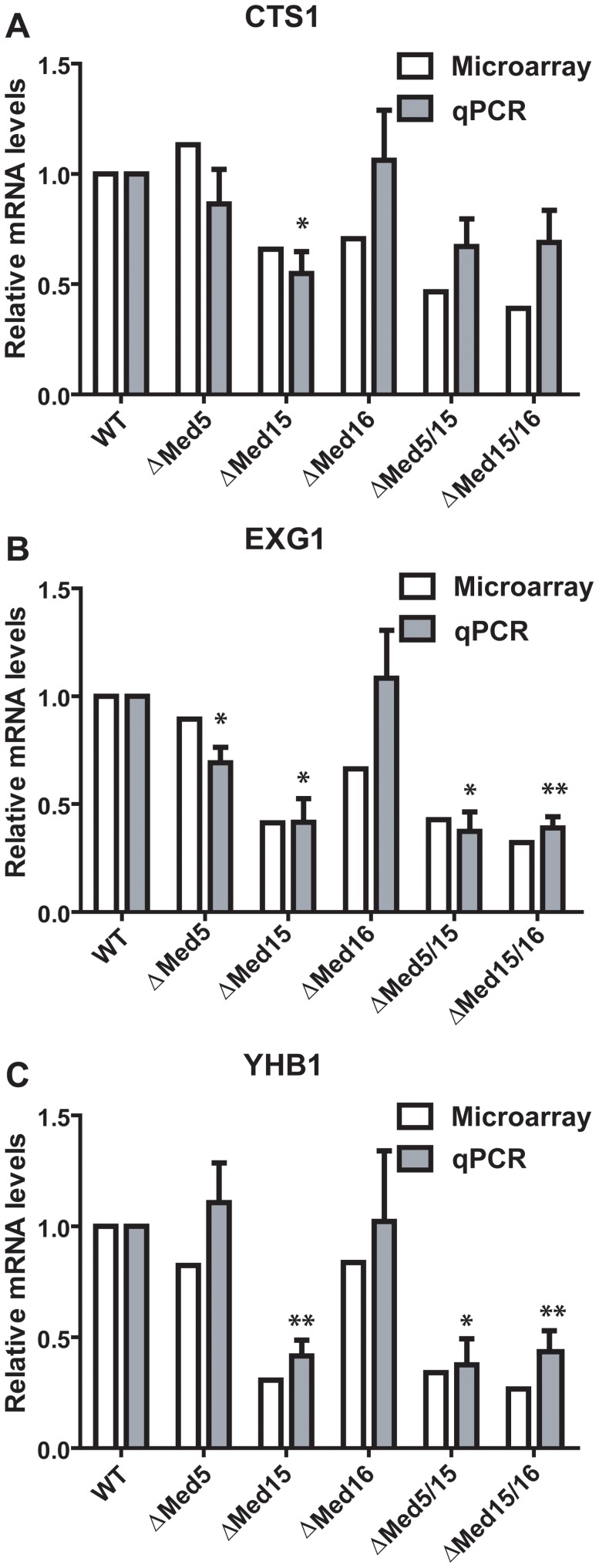
Confirmation of Ace2 target genes. mRNA levels of the genes CTS1, EXG1 and YHB1 from WT and Degron-strains were measured using qPCR and normalized against the WT level. qPCR levels are compared to the levels determined in the corresponding microarray assays. The experiments were performed in biological triplicates, and error bars represent the standard deviation. P-values where calculated using Student’s t-test. * Indicates p-value < 0.05, ** indicates p-value <0.01.

*DSE1* and *SCW11,* which are specifically down regulated in the *med5/med15* double Degron strain (**Table 1**), have both been identified as two of a total of 22 targets genes for the transcriptional activator Ace2 [33], [34]. In order to study the potential involvement of Ace2 in the synthetic lethality, we examined our microarray data in more detail. Remarkably, we found that 11 of the 22 previously identified Ace2 target genes were significantly down regulated in both the *med5/med15* and *med15/med16* double Degron mutants when compared only to the wild-type strain (**Table 2**). Several of these genes (i.e. *CLN3*, *CTS1*, *AMN1*, *EGT2*, *DSE1*, *SCW11*) have been shown to be induced in G1 and are required for normal progression through G1 [35]–[40]. The reason why these genes were not initially scored as uniquely regulated in the double Degron strains is that several genes that were significantly down regulated in the double Degron strains were down regulated to some extent already in the *med15* single Degron strain. Down-regulation of three of the Ace2-target genes was verified using qPCR (**Figure 4**). One obvious explanation for this finding is that Ace2 is itself affected by loss of Med15. However, we found no effects of changes in Ace2 expression in any of our Degron strains and we thus conclude that the observed effects are most likely caused by a requirement of Med15 for the ability of Ace2 to affect its target promoters, rather than a requirement of Med15 for expression of *ACE2*. Irrespective of mechanism, our results indicate that the Med15 subunit is required for activation of genes in the Ace2 pathway.

**Table 2:** Changes in expression levels of genes that are identified as target genes for the transcription factor Ace2 in the *med5/med15*, *med15/med16* and *med15* Degron strains relative to wild type cells.

Gene name	Protein	Protein function	Fold down in		
			med5/15vs. Wt	med15/16vs. Wt	med15vs. WT
YAL040C	CLN3	G1 cyclin involved in cell cycle progression; activates Cdc28p kinase to promote the G1 to S phase transition; plays a role in regulating transcription of the other G1 cyclins, CLN1 and CLN2; regulated by phosphorylation and proteolysis	1.8	2.0	1.6
YNL327W	EGT2	Glycosylphosphatidylinositol (GPI)-anchored cell wall endoglucanase required for proper cell separation after cytokinesis, expression is activated by Swi5p and tightly regulated in a cell cycle-dependent manner	1.6	1.8	1.4
YLR286C	CTS1	Endochitinase, required for cell separation after mitosis; transcriptional activation during the G1 phase of the cell cycle is mediated by transcription factor Ace2p	1.7	1.9	1.4
YLR300W	EXG1	Major exo-1,3-beta-glucanase of the cell wall, involved in cell wall beta-glucan assembly; exists as three differentially glycosylated isoenzymes	1.8	2.2	1.8
YBR157C	ICS2	Protein of unknown function	2.0	2.5	2.2
YGR234W	YHB1	Nitric oxide oxidoreductase, flavohemoglobin involved in nitric oxide detoxification; plays a role in the oxidative and nitrosative stress responses	2.1	2.5	2.3
YBR158W	AMN1	Protein required for daughter cell separation, multiple mitotic checkpoints, and chromosome stability; contains 12 degenerate leucine-rich repeat motifs; expression is induced by the Mitotic Exit Network (MEN)	2.2	2.1	2.0
YJL078C	PRY3	Protein of unknown function	2.0	2.2	1.9
YGL028C	SCW11	Cell wall protein with similarity to glucanases; may play a role in conjugation during mating based on its regulation by Ste12p	3.8	3.6	2.4
YER124C	DSE1	Daughter cell-specific protein, deletion affects cell separation after division and sensitivity to drugs targeted against the cell wall	2.2	2.1	1.4
YJL157C	FAR1	Cyclin-dependent kinase inhibitor that mediates cell cycle arrest in response to pheromone			

### Depletion of Med15, Med5/Med15 or Med15/Med16 causes cell cycle defects

**Figure 5:**
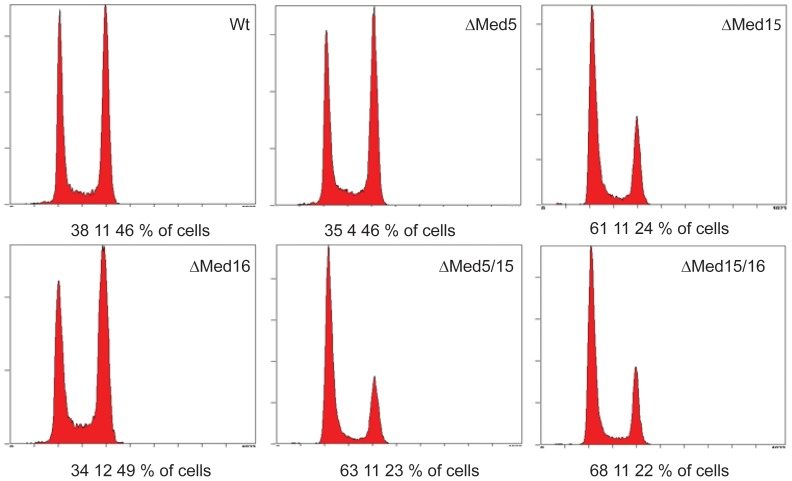
Flow cytometry analyses of Degron constructs. DNA content of cells carrying the indicated Degron constructs was analyzed by flow cytometry at 3 hours after switching from the permissive to the restrictive growth conditions. Numbers below each histogram indicate the percentage of cells in the G1-, the S-, and the G2+M-phases, respectively.

Several of the genes that we identified as down regulated in the *med15* single Degron strain and the *med5/med15* and *med15/med16* double Degron strains, encode proteins that function in cell wall organization, cell wall biogenesis and cell division (**Tables 1** **and S1**). Notably, the Ace2 transcription factor described above is well known for its involvement in cell cycle regulation [41]. We therefore used flow cytometry analyses to study if these strains showed any cell cycle defects. At 3 hours after switching from the permissive conditions to the restrictive conditions, we found that all three strains expressing the *MED15* Degron construct behaved differently from the strains that do not contain the *MED15* Degron (**Figure 5**). More specifically, in the three strains that contain the *MED15* Degron the fraction of cells in the S/G2 phases was only 22–24%, compared to more than 50% in the strains with normal Med15 expression. This indicates that loss of Med15 leads to a delay in cell cycle progression by arresting in G1 phase [42]. Interestingly, this correlates well with a previous report demonstrating that Ace2 activates transcription of G1 specific genes [34]. These results suggest that the requirement of Med15 for Ace2-mediated transcriptional activation may be reflected by the observed cell cycle defects in the strains expressing Degron-tagged Med15.

## Discussion

In contrast to the well described structure of Mediator, and the knowledge we have gained from vast number of reports on the consequences of deletion of genes that encode Mediator subunits, very little is known about the molecular mechanisms for Mediator function in transcriptional regulation. Some favor a recruitment model, where transcriptional activators are bound to specific promoter sequences in response to environmental cues transmitted via different signal transduction pathways. The promoter-bound transcriptional activators are then suggested to operate by recruiting Mediator and subsequently the general transcription machinery including Pol II to the promoter which then becomes activated (see [43] for a review). Other reports support a model where promoter-bound transcriptional regulators induce conformational changes in Mediator that are in turn propagated to the general transcription machinery [44], [45]. In addition, there are several reports of post-translational modifications of both transcriptional activators (*i.e.* Gal4 [46], [47], Mediator subunits (*i.e.* Med2 [24], [25], and general transcription factors (*i.e.* TFIIF [48]). It seems likely that recruitment, induced conformational changes and post-translational modifications of Mediator and general transcription factors are sufficient to explain the basic principles for transcriptional regulation and the ability to integrate signals from different combinations of activators and repressors that are bound concomitantly to one promoter, but fine-tuning of gene expression might also rely on other mechanisms.

We and others have shown that some genes that encode Mediator tail subunits, *i.e. MED5* and *MED15* or *MED15* and *MED16* are synthetically lethal [26], [27]. However, it is difficult to reveal the primary cause of this lethality using regular double deletion mutants since these cells are inviable. Previous experiments on single deletion mutants of these subunits have shown that they display specific non-lethal phenotypes. *MED16* deletion mutants are viable but show some growth defects when grown under normal conditions including an abnormal elongated shape, flocculation, heat sensitivity and failure to enter stationary phase upon nutrient deprivation[49]. *MED15* single deletion mutants are also viable in normal growth conditions. However, several reports have shown that *med15* cells are affected under a number of specific growth conditions i.e. fatty acid media [50], decreased osmotic stress resistance, and growth defect at high salt concentrations [51], decreased resistance to oxidative stress [52]. It is also reported that sporulation efficiency is decreased in *med15* [53]. However, most of these studies are large-scale surveys and not specifically aimed at Med15. Finally, single *med5* mutants are viable under normal growth conditions but show increased respiration (grows better than wild type on the nonfermentable carbon source glycerol), display an increased oxygen consumption and influences expression of nuclear and mitochondrial OXPHOS genes [27].

In order to gain a broader understanding of the causes for the synthetic lethality of *MED5/MED15* and *MED15/MED16*, we adopted and developed the N-Degron system in order to make it possible to study the immediate effect on gene expression shortly after inactivation of two essential genes. We found that double Degron strains of *MED5*/*MED15* or *MED15/MED16* both showed upregulation of a set of genes involved in meiosis and sporulation. This could be a result of the down regulation of *RCK1*, which we observed in the double Degron strains. Deletion of *RCK1* was previously shown to increase the rate of sporulation and it is therefore believed to be a repressor of meiosis [32]. On the other hand, the up regulation of meosis and sporulation genes in the double Degron strains might be the result of derepression, directly caused by the conditional deletion of *MED5/MED15* or *MED15/MED16*. We hypothesize that a number of genes that normally need to be repressed in haploid cells are derepressed in the double Degron strains and a situation of ‘ectopic meiosis ‘occurs. This event might lend an explanation for the observed synthetic lethality.

Expression profiling of the Degron strains also revealed that several target genes of the Ace2 transcriptional activator were immediately downregulated in response to *MED15* inactivation. In the budding yeast *Saccharomyces cerevisiae*, Ace2 controls expression of factors that are required for septum destruction after cytokinesis and it has been shown that mutant Ace2 protein causes a G1 phase delay [54]. It is therefore interesting that we found that all Degron strains that express Med15 as a Degron construct (*med15*, *med5/med15*, and *med15/med16*) show a G1 delay at the restrictive conditions (**Figure 5**). Similar results have also been reported for an *ace2* mutant in fission yeast [55]. Since we could not detect significant changes in expression of Ace2 itself in any of these strains, we conclude that the observed effects are caused by the absence of Med15. We postulate that Med15 transmits regulatory signals from Ace2 to the Pol II transcription machinery.

Interestingly, much of the transcriptional de-regulation observed in the double Degron strains was also apparent in the *med15* single Degron strain. This was also supported by our finding that inactivation of *MED15* using the Degron system causes a clear temperature senstive phenotype on plates, and lethality when the cells are grown in liquid cultures. In summary, our study has identified transcriptional targets of the Mediator Tail module, thereby providing new insight into the mechanisms underlying Mediator function.

## Supporting Information

Table S1 **Comparisons of expression levels between wild type cells, single Degron mutants and double Degron mutants.**
(XLS)

Table S2 **Genes up or downregulated in the Med5/Med15 or Med15/1Med16 strains and are involved in meiosis or sporulation.**
(DOCX)
