# Supplementary material for: Functional Studies of the Yeast Med5, Med15 and Med16 Mediator Tail Subunits
Source: PLoS One. 2013 Aug 22;8(8):e73137. doi: 10.1371/journal.pone.0073137 (PMC3750046; doi:10.1371/journal.pone.0073137)
Supplement: Table S2 — Genes up or downregulated in the Med5/Med15 or Med15/1Med16 strains and are involved in meiosis or sporulation. (DOCX) [file pone.0073137.s002.docx]

| **Gene Name** | **Gene** | **Change in Med5/15** | **Change in**  **Med15/16** | **References** | **Function** |
| --- | --- | --- | --- | --- | --- |
| *Downregulated* | | | | | |
| YGL158W | RCK1 | ↓ | ↓ |  | Protein kinase involved in the response to oxidative stress and in inhibition of meiosis |
| *Upregulated* | | | | | |
| YGL033W | HOP2 | ↑ | ↑ | [1,2] | Meiosis-specific protein that localizes to chromosomes, preventing synapsis between non-homologous chromosomes and ensuring synapsis between homologs; complexes with Mnd1p to promote homolog pairing and meiotic double-strand break repair |
| YLR341W | SPO77 | ↑ | ↑ | [3] | Meiosis-specific protein of unknown function required for spore wall formation during sporulation |
| YOL132W | GAS4 | ↑ | ↑ | [4] | 1,3-beta-glucanosyltransferase, involved in spore wall assembly, localizes to the cell wall |
| YFL012W | NA | ↑ | ↑ | [5] | Putative protein of unknown function, transcribed during sporulation |
| YDR446W | ECM11 | ↑ |  | [6] | Protein apparently involved in meiosis, may be involved in maintaining chromatin structure |
| YPL130W | SPO19 | ↑ |  | [7,8] | Meiosis-specific prospore protein; required to produce bending force necessary for proper assembly of the prospore membrane during sporulation |
| YOR242C | SSP2 | ↑ |  | [9,10] | Sporulation specific protein that localizes to the spore wall, required for sporulation at a point after meiosis II and during spore wall formation |
| YDR218C | SPR28 | ↑ |  | [11] | Sporulation-specific homolog of the yeast CDC3/10/11/12 family of bud neck microfilament genes; meiotic septin expressed at high levels during meiotic divisions and ascospore formation |
| YHR185C | PFS1 |  | ↑ | [12] | Sporulation protein required for prospore membrane formation at selected spindle poles |

**Table S2: Genes up or downregulated in the Med5/Med15 or Med15/1Med16 strains and involved in meiosis or sporulation.**

**Supplementary references**

1. Leu JY, Chua PR, Roeder GS (1998) The Meiosis-Specific Hop2 Protein of S. cerevisiae Ensures Synapsis between Homologous Chromosomes. Cell 94: 375–386.

2. Henry JM, Camahort R, Rice DA, Florens L, Swanson SK, et al. (2006) Mnd1/Hop2 facilitates Dmc1-dependent interhomolog crossover formation in meiosis of budding yeast. … and cellular biology 26: 2913–2923. doi:10.1128/MCB.26.8.2913-2923.2006.

3. Rabitsch KP, Tóth A, Gálová M, Schleiffer A, Schaffner G, et al. (2001) A screen for genes required for meiosis and spore formation based on whole-genome expression. Curr Biol 11: 1001–1009.

4. Ragni E, Coluccio A, Rolli E, Rodriguez-Peña JM, Colasante G, et al. (2007) GAS2 and GAS4, a pair of developmentally regulated genes required for spore wall assembly in Saccharomyces cerevisiae. Eukaryotic … 6: 302–316. doi:10.1128/EC.00321-06.

5. Naitou M, Hagiwara H, Hanaoka F, Eki T, Murakami Y (1997) Expression profiles of transcripts from 126 open reading frames in the entire chromosome VI of Saccharomyces cerevisiae by systematic northern analyses. Yeast 13: 1275–1290. doi:10.1002/(SICI)1097-0061(199710)13:13<1275::AID-YEA172>3.0.CO;2-7.

6. Zavec AB, Lesnik U, Komel R, Comino A (2004) The Saccharomyces cerevisiae gene ECM11 is a positive effector of meiosis. FEMS Microbiol Lett 241: 193–199. doi:10.1016/j.femsle.2004.10.020.

7. Primig M, Williams RM, Winzeler EA, Tevzadze GG, Conway AR, et al. (2000) The core meiotic transcriptome in budding yeasts. Nature 26: 415–423. doi:10.1038/82539.

8. Tevzadze GG, Pierce JV, Esposito RE (2007) Genetic evidence for a SPO1-dependent signaling pathway controlling meiotic progression in yeast. Genetics 175: 1213–1227. doi:10.1534/genetics.106.069252.

9. Li J, Agarwal S, Roeder GS (2006) SSP2 and OSW1, Two Sporulation-Specific Genes Involved in Spore Morphogenesis in Saccharomyces cerevisiae. Genetics 175: 143–154. doi:10.1534/genetics.106.066381.

10. Sarkar PK, Florczyk MA, McDonough KA, Nag DK (2002) SSP2, a sporulation-specific gene necessary for outer spore wall assembly in the yeast Saccharomyces cerevisiae. Mol Genet Genomics 267: 348–358. doi:10.1007/s00438-002-0666-5.

11. De Virgilio C, DeMarini DJ, Pringle JR (1996) SPR28, a sixth member of the septin gene family in Saccharomyces cerevisiae that is expressed specifically in sporulating cells. Microbiology (Reading, Engl) 142 ( Pt 10): 2897–2905.

12. Enyenihi AH, Saunders WS (2003) Large-scale functional genomic analysis of sporulation and meiosis in Saccharomyces cerevisiae. Genetics 163: 47–54.
